# Biocontrol Potential of *Sclerotinia sclerotiorum* and Physiological Changes in Soybean in Response to *Butia archeri* Palm Rhizobacteria

**DOI:** 10.3390/plants9010064

**Published:** 2020-01-03

**Authors:** Luciana Cristina Vitorino, Fellipe Oliveira da Silva, Bárbara Gonçalves Cruvinel, Layara Alexandre Bessa, Márcio Rosa, Edson Luiz Souchie, Fabiano Guimarães Silva

**Affiliations:** 1 Laboratory of Agricultural Microbiology, Federal Institute Goiano Rio Verde Campus, Rodovia Sul Goiana, Km 01, 75901-970 Rio Verde-GO, Brazil; fellipeoogleplay92@gmail.com (F.O.d.S.); barbaracruvinel.rv@gmail.com (B.G.C.); edson.souchie@ifgoiano.edu.br (E.L.S.); 2 Laboratory of Plant Mineral Nutrition, Federal Institute Goiano Rio Verde Campus, Rodovia Sul Goiana, Km 01, 75901-970 Rio Verde-GO, Brazil; layara.bessa@ifgoiano.edu.br (L.A.B.); fabiano.silva@ifgoiano.edu.br (F.G.S.); 3 Rio Verde University (UniRV)-Rio Verde Campus, Fazenda Fontes do Saber, Caixa Postal 104, 75901-970 Rio Verde-GO, Brazil; marcio1506-@hotmail.com

**Keywords:** biological control, Cerrado, promotion of plant growth, rhizospheric bacteria

## Abstract

*Sclerotinia sclerotiorum* is a necrotrophic parasitic fungus that causes *Sclerotinia* stem rot (SSR), which is currently one of the most difficult agronomic crop diseases to control. A number of plants of the Brazilian Cerrado biome have been shown to be important sources of symbiotic microorganisms with biotechnological potential, so we decided to test the potential of bacteria isolated from the dwarf jelly palm, *Butia archeri* (Arecaceae) for the control of the pathogenic effects provoked by *S. sclerotiorum*. For this, we bioprimed seeds and evaluated the effects of this biopriming on the OJIP transient patterns prior to and following infection by the phytopathogen. Plants treated with the BA48R strain of *Enterobacter* sp., and in particular, those treated with the BA88R strain of *Bacillus cereus* presented the best results in terms of the loss/gain of the physiological and symptomatological variables evaluated. The plants bioprimed with BA88R presented high post-infection levels of total chlorophyll (33.35 FCIs) and chlorophyll *a* (26.39 FCIs), maintained a high Nitrogen Balance Index (NBI = 18.87), and synthesized low concentrations of flavonoids (1.39). These plants also maintained high levels of PI_ABS_ (1.111) and PI_TOTAL_ (1.300) following infection, and low levels of Di_0_/RC (0.602), which indicates that, in the presence *S. sclerotiorum*, the efficiency of the photosynthesis in the plants treated with these bacteria was less affected in the reaction centers, as confirmed by the negative amplitude recorded in the L band. The present study reconfirms the importance of the use of chlorophyll fluorescence for the diagnosis of disease and conditions of stress in crop plants, in addition to demonstrating the effectivenesss of the BA48R bacterial strain and, in particular, the BA88R strain on systemic resistance induction and suppression of *S. sclerotiorum* in *Glycine max* plants, with enormous potential for the development of more sustainable agricultural processes.

## 1. Introduction

The coexistence of plants and microorganisms in the natural environment has led to the establishment of both negative and positive interactions between different species. Plants and microorganisms compete for access to the resources that guarantee their quality of life, which has led to the evolution of countless adaptive strategies, such as the resistance mechanisms in the plants and pathogenic lifestyles in the microbiota [1]. Plants have an enhanced capacity for the recognition of pathogens, through strategies that involve both conserved elicitors and the characteristics of the pathogens themselves, while pathogens are capable of overcoming physical barriers, suppressing or avoiding immunological mechanisms, and obtaining nutrients from the tissue of the host plant. To achieve this, the microorganisms secrete virulence effector molecules [2,3]. In other cases, the plants and microorganisms may both derive advantages from their coexistence. Elicitation of induced systemic resistance (ISR) by rhizospheric bacteria, for example, was initially reported using *Pseudomonas* spp. [4], but currently many other species show elicitor potential e.g., [5]. Bakker et al. [6] explain that the efficacy of these bacteria in biological control lies in combining ISR, through the synthesis of salicylic acid, for example, with direct antibiosis, through the production of siderophores or antimicrobials.

A number of plants of the Brazilian Cerrado biome have been shown to be an important source of microorganisms with biotechnological potential e.g., [7,8,9]. Silva et al. [10,11] recently isolated rhizospheric and endophytic of the palm *Butia purpurascens* (Arecaceae), and evaluated the multifunctional potential of these microorganisms, many of which can be used as plant growth promoters. The dwarf jelly palm, *Butia archeri* Glassman, known as the “coqueirinho-do-campo” or “butiá” in its native Brazil, is a short-trunked palm endemic to the Cerrado savanna biome, and is thus well-adapted to the soils of this environment. *Butia archeri* is found only in the Brazilian states of Goiás, Minas Gerais, and São Paulo, as well as the Federal District [12,13]. Despite being listed as extinct in the threatened species red-list of the state of São Paulo [14], *Butia archeri* is locally abundant in midwestern Brazil, i.e., Goiás and the Federal District. Vitorino et al. [15] recently demonstrated that rhizospheric isolates of this plant can promote the health of soybean seeds by suppressing the development of spoilage fungi such as *Colletotrichum truncatum*, *Aspergillus flavus* and *Penicillium* sp. Based on these findings, and the fact that the rhizospheric community of this plant have yet to be evaluated sufficiently, we decided to test the hypothesis that the rhizospheric bacteria of this plant may promote growth and suppress the damaging effects of the phytopathogenic fungus *S. sclerotiorum*. To test this hypothesis, we evaluated the biocontrol potential of these bacteria in soybean, *Glycine max* (L.) Merr., plants.

Soybean plants are often affected by *S. sclerotiorum*, a necrophytic parasite [16] that causes *Sclerotinia* stem rot (SSR). In the specific case of soybean, SSR can limit the productivity of the plant severely under favorable environmental conditions [17]. The affected plants produce fewer, smaller seeds [18,19] as a result of the ringing of the stem and the rupturing of the xylem and phloem. A loss of the germinative capacity of the seeds and a decrease in the production of oil have also been recorded [20]. Given the lack of resistant cultivars or any practical or cost-effective control measures [21], this fungus has had increasingly negative impacts on soybean crops. In the United States, SSR was included among the top 10 pathogens that reduced the productivity of the soybean crop in 2000, 2004, 2006, and 2009, and it continues to have a significant impact on the productivity of this oilseed in the present day [22,23]. Between 2010 and 2014, SSR was responsible for the loss of almost 2.8 million tons of soybean, with a market value of US$1.2 billion [22,24].

*Sclerotinia* stem rot has also become a major agricultural problem in other soybean producing regions, such as Brazil, where the loss of productivity may reach 60% in some cases [25]. The integrated management of SSR is based on a combination of cropping, chemical, and biological controls. In most cases, however, these practises are adopted in an unsystematic fashion, which reduces their effectiveness. Given this, the application of chemical fungicides, such as picoxystrobin (Aproach^®^) and boscalid (Endura^®^), is the standard procedure used for the control of SSR [26,27]. Fungal biological controllers, such as *Trichoderma asperelloides*, *Trichoderma harzianum*, and *Coniothyrium minitans* e.g., [28,29,30], have been considered to be a viable alternative for the treatment of soybean seed, although alterations in the water potential of the soil may limit the use of these fungi for biocontrol [31]. Even so, the ongoing advances in the development of new microbial strains with potential for biological control provide increasing hope for the discovery of new, as yet untested strains that are more effective for the suppression of *Sclerotinia*.

The infectious mechanism of *S. sclerotiorum* consists of the synthesis of oxalic acid, which acts as a virulence factor, inducing programmed cell death (PCD) in the host plant, a process that depends on the generation of reactive types of oxygen produced primarily by NADPH oxidases located in the plasmatic membrane of the plant cell [16]. Previous studies have shown that the efficiency of photosynthesis is reduced in plants infected with *S. sclerotiorum*. This photosynthetic depletion is induced by a significant reduction in the maximum quantic production of photosystem (PS) II (F_v_/F_m_) e.g., [32,33]. The reaction centers of the PS II are damaged severely by *S. sclerotiorum* infection e.g., [34]. Based on this scenario, we decided to test the potential of rhizospheric bacteria for the suppression of the symptoms of *S. sclerotiorum* infection in *G. max* plants, based on the evaluation of the stress provoked by the pathogenesis, alterations in leaf pigments, modifications of the OJIP chlorophyll fluorescence kinetics, and the appearance of symptoms of leaf necrosis.

## 2. Material and Methods

### 2.1. Bacterial Isolates and the Phytopathogenic Strain

The present study evaluated the potential of 15 rhizopheric bacterial isolates for the biocontrol of a phytopatogenic strain of *S. sclerotiorum* obtained from the micro-organism collection of the Agricultural Microbiology Laboratory of the Rio Verde campus of the Goiás Federal Institute, in the Brazilian state of Goiás. The bacterial isolates were obtained from the rhizophere of *Butia archeri* (Silva et al. in press) and the phytopathogenic strain was isolated from soybean plants with symptoms of SSR. The bacteria and the phytopathogenic fungus were identified by their 16S rDNA and 18S rDNA gene sequences (respectively) through a BLAST search of GenBank. The strains were maintained permanently in 10% glycerol in a freezer at −80 °C. After defrosting, the bacteria were reactivated in Nutrient Agar-NA (3 g of meat extract, 5 g of peptone, 25 g of agar, and H_2_O q.s. 1 L) for 48 h at 30 °C in a growth incubator, while the phytopathogen was reactivated in potato dextrose agar (PDA) medium (infusion of 200 g of potato, 20 g of dextrose, 15 g of agar, and H_2_O q.s. 1 L), incubated at 25 °C for five days.

### 2.2. Preparation of the Biocontrollers and Inoculation of the Seeds

The soybean seeds used in this study were of the Syn 13610 IPRO^®^ cultivar. The seeds were de-infested superficially for the removal of the epiphytes through repeated rinses under running water followed by agitation in water and neutral detergent at 70 rpm for 10 min. After successive rinses, the seeds were immersed in 70% ethanol for 1 min, sodium hypochloride (2.5% of active chlorine) for 5 min, and then 70% ethanol for 30 s, followed by three rinses with autoclaved distilled water.

The bacterial inoculates were prepared in NA broth for 24 h at 30 °C, under constant agitation (90 rpm) in an orbital agitator. The concentration of cells in each culture was then estimated by counting the CFUs in NA medium. The concentration of cells recorded in each culture was standardized to 10^7^ mL^−1^, using a 0.85% saline solution. The soybean seeds were bioprimed separately with each bacterial strain, with 30 seeds being bioprimed per strain. For this, the seeds were immersed for 20 min under constant agitation (50 rpm) in an orbital agitator. For the control treatment, seeds were immersed in the pure culture medium, with no inoculant. The seeds were separated from the bacterial broth used for each treatment prior to planting.

### 2.3. Plant Maintenance and Inoculation with the Phytopathogen

The phytopathogenetic assays were conducted between September and December, 2018, in the greenhouse of the Plant Tissue Culture Laboratory on the Rio Verde campus of the Goiás Federal Institute in Goiás, Brazil. The seeds were planted in 5-L pots filled with 4 L of soil as the growing substrate. This substrate was dystrophic red latosol, with the following chemical parameters in the 0–20 cm layer: pH (CaCl_2_) = 5.7, O.M. = 23.0 g·kg^−1^, P = 14 mg·dm^−3^, K = 0.409 mmolc·dm^−3^, Ca = 4.0 mmolc·dm^−3^, Mg = 0.9 mmolc·dm^−3^, and H + Al = 1.8 mmolc·dm^−3^. The soil was analyzed at the Rio Verde campus of the Goiás Federal Institute, where it was sieved through a 2 mm mesh and then autoclaved at 121 °C for 30 min. The sterilization of the soil and the asepsis of the seed surface were applied to eliminate any microorganisms that might compete with the inoculates in the colonization of the plant tissue.

A total of 10 soybean seeds were planted per pot, with the seedlings being thinned at the VC (cotyledon) stage, leaving only two plants per pot. These plants were watered daily.

The soybean plants were inoculated with the phytopathogenic strain of *S. sclerotiorum* 65 days after planting. For this, the mycelium was cultured in Brain Heart Infusion (BHI) broth for 7 days at 30 °C, under constant agitation (90 rpm) in an orbital agitator. The increase in the concentration of the spores was monitored by counting in a Neubauer chamber (hemacytometer) under a light microscope (magnification of 40–100×) with inoculation being initiated when the estimated concentration reached 10^7^ spores/mL. The fourth leaf of each soybean plant was sprayed with the spore broth and monitored for symptoms daily for the subsequent 7 days.

### 2.4. Physiological Analyses

The physiological data were obtained from the fourth leaf of each plant, at two stages: pre- and post-inoculation with the pathogen (referred to here as “pre-pathogen” and “post-pathogen”). This was important to differentiate the potential growth-promoting effects of the rhizospheric bacteria tested in the present study from the modified physiological responses resulting from the attack of the pathogen. The pre-pathogen assessments were conducted at 60 days after the seeds were planted, while the post-pathogen assessments were conducted 72 days after planting.

The chlorophyll (*a*, *b*, and total) indices for the leaf tissue were determined by a portable ClorofiLOG1030^®^ meter (Falker^®^, Porto Alegre, RS, Brazil). The values were expressed as the Falker chlorophyll index (FCI).

The flavonoid and anthocyanin indices were also determined, as well as the Nitrogen Balance Index (NBI), using a Dualex Scientific^TM^ sensor (Force-A, Orsay, France), based on the chlorophyll fluorescence excitation spectra [35]. The NBI was estimated from the ratio between the chlorophylls and flavonoids. The pigments were evaluated by taking readings between 09:00 h and 11:00 h.

The OJIP transient fluorescence of the chlorophyll *a* was determined using a portable FluorPen FP 100 fluorometer (Photon Systems Instruments; Drasov, Czech Republic). The fourth leaf of each sample unit (plant) was first adapted to the dark for 30 min, for the complete oxidation of the photosynthetic electron transportation system. Each leaf was then submitted to a pulse (3000 µmol·m^−2^·s^−1^) of blue light, with the minimum fluorescence (Fo) being measured at 50 μs, when all the Photosystem II (PSII) reaction centers are open. This was defined as step O, and was followed by step J (at 2 ms), step I (at 30 ms), and the maximum fluorescence (Fm), when all the PSII reaction centers are closed, as step P. These values were used to estimate the different bioenergetic indices of the PSII, following Strasser et al. [36]. The transient fluorescence data were used to estimate the parameters established by the JIP test. The maximum efficiency of the PSII (F_V_/F_M_) was also estimated, as was the partial performance index (PI_ABS_), which encompasses the energy cascade from the initial absorption events to the reduction of the PQ. The specific energy dissipation flow of the chlorophyll of the antenna complex (DI_0_/RC) was also measured, as was the general photosystem performance index (PI_TOTAL_), which measures the performance up to the final electron receptors of Photosystem I (PSI).

Parameters related to leaf area were also evaluated: area suffering necrosis as a result of the SSR and percentage of leaf area corresponding to necrotic area. This parameter was obtained through the analysis of the fourth leaf, with the ImageJ image-treating software (National Institutes of Health, Bethesda, MD, USA) being used to measure the area [37].

### 2.5. Experimental Design and Statistical Analyses

The experiment was based on an entirely randomized design, and the physiological analyses were run in a double factorial layout (15 bacteria + 1 control × two evaluation stages-pre- and post-pathogen). The data were thus compared among the different bacterial treatments and between pre- and post-pathogen stages. The treatments were evaluated in triplicate, with each pot containing two plants being considered a sample unit. The physiological data were tested for normality, and an Analysis of Variance (ANOVA) was applied. The significance of the differences between pairs of means was determined using the Scott Knott test, with a 5% probability level. The statistical analyses were run in the R environment, version 3.4.3. [38].

## 3. Results

### 3.1. Physiological Analyses of Pigments

In the pre-pathogen plants, the highest mean chlorophyll *a* index (FCI = 30.63) was recorded in the plants treated with *Bacillus cereus* strain BA81R. The plants treated with the BA80R, BA103R, and BA110R strains of *Enterobacter* sp. (FCI = 29.30, 28.35, and 27.37, respectively) and the control treatment (FCI = 29.08) also accumulated relatively high levels of chlorophyll.

No major differences were observed among the treatments in the chlorophyll *b* indices, although the total chlorophyll index followed the same pattern as chlorophyll *a*, with the highest mean value (FCI = 40.48) being recorded in the BA81R treatment (Table 1).

Following inoculation with *S. sclerotiorum*, the plants bioprimed with *B. cereus* strains BA81R and BA88R, *Enterobacter* sp. strains BA80R and BA110R, *Bacillus* sp. strain BA122R, and *Enterobacter asburiae* strain BA123R presented the highest chlorophyll *a* indices (FCI = 26.93, 26.39, 26.21, 26.72, 27.03, and 26.55, respectively). In the case of the chlorophyll *b*, the highest indices were recorded in the plants treated with strains BA81R (FCI = 7.42), BA110R (FCI = 7.35), and BA122R (FCI = 7.68). As observed in the pre-pathogen group, the total chlorophyll followed the same pattern as the chlorophyll *a*, with the highest indices being recorded in the plants treated with *B. cereus* strains BA81R and BA88R, *Enterobacter* sp. strains BA80R and BA110R, BA122R (*Bacillus* sp.), and the BA123R strain of *E. asburiae* (Table 1).

Inoculation with the pathogen resulted in a decrease in the chlorophyll index of the leaves of the *G. max* plants in most of the bacterial treatments. However, the greatest reduction was observed in the control group, in which chlorophyll *a* decreased by 25.48%, chlorophyll *b* by 46.87%, and total chlorophyll by 30.98%. Unexpectedly, a number of bacterial treatments presented an increase in the chlorophyll *a* index following inoculation with the pathogen, including the BA45R strain of *Pantoea cypripedii* (an increase of 2.36%), *B. cereus* strain BA88R (6.41%), *Bacillus* sp. strain BA122R (5.26%), and *E. asburiae* strain BA123R (5.70%). This resulted in an increase of 3.68%, 2.86%, 2.63%, and 3.48%, respectively, in the total chlorophyll index in these treatments, which indicates that the presence of these symbiotic bacteria in the plant tissue reduced the effects of the stress caused by the pathogen.

### 3.2. Physiological Analyses of Nitrogen Balance, Flavonoid and Anthocyanin Indices

The highest nitrogen balance indices were recorded in the plants treated with *B. cereus* strain BA81R (NBI = 27.83), *Enterobacter* sp. strain BA103R (NBI = 29.10), and *Enterobacter oryzae* strain BA106R (NBI = 28.90), with these values being related invariably to high concentrations of chlorophyll and low concentrations of flavonoids (Table 2). Given this, the lowest flavonoid indices were recorded in the plants treated with the BA81R (FLAV = 1.16), BA103R (FLAV = 1.03), and BA106R (FLAV = 1.03) bacterial strains. The mean anthocyanin levels recorded in the pre-pathogen group were similar across all treatments.

Following inoculation with *S. sclerotiorum*, the highest NBI value (18.87) was recorded in the plants treated with *B. cereus* strain BA88R. This value coincided, once again, with the highest total chlorophyll concentration and the lowest flavonoid concentration (FLAV = 1.39). The plants of this treatment also synthesized low concentrations of anthocyanins (ANTH = 0.29). The highest mean flavonoid index (FLAV = 1.57) was recorded in the plants treated with *E. asburiae* strain BA123R, although these plants also had low concentrations of anthocyanins (ANTH = 0.29), as did the plants in the control treatment (Table 2).

In general, the NBI values were reduced following the stressing contact of the plants with the pathogen, which indicates a relative increase in the concentration of flavonoids in comparison with that of chlorophyll in the leaves. However, a less drastic reduction (only 19.01% in comparison with the pre-pathogen values) was observed in the plants treated with *B. cereus* strain BA88R, which indicates that the presence of this bacterium may capacitate the plant to maintain its physiological homeostasis in the presence of *S. sclerotiorum*.

A general increase in the production of flavonoids was observed when the plants were attacked by the pathogen, with most treatments presenting higher flavonoid concentrations than the control plants. In the control plants, the flavonoid index increased by only 18.4%, whereas in the plants treated with *Enterobacter* sp., this index increased by 34.95% in the case of the BA80R strain and 31.89% in BA103R, the greatest percentage increase in flavonoid concentrations recorded in the study, followed by *E. asburiae* strain BA123R, at 29.95%. The plants treated with *B. cereus* strain BA88R not only maintained their NBI levels, but also presented the smallest modifications in the levels of chlorophyll (a reduction of 19.47%), flavonoids (an increase of 15.83%), and anthocyanins (an increase of 7.40%).

### 3.3. OJIP Fluorescence Transient Analysis

**Figure 1:**
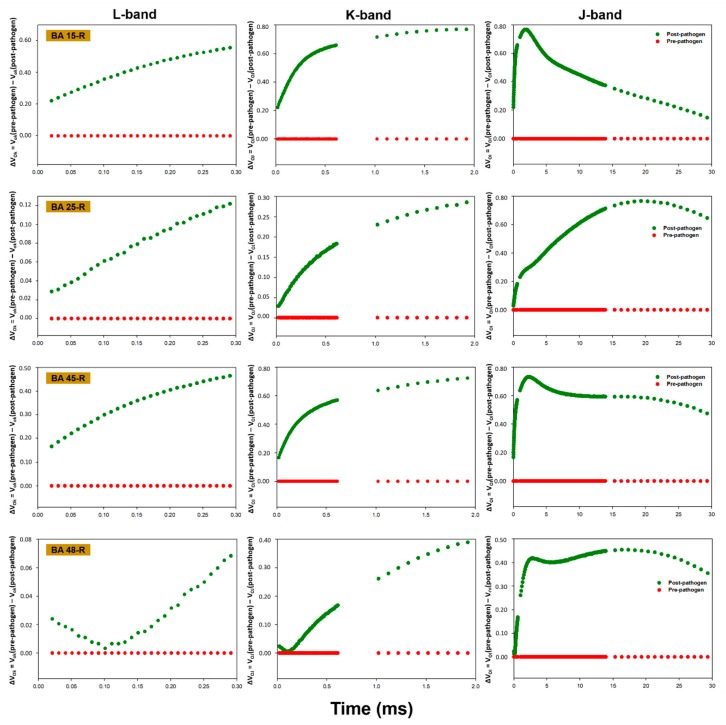
Kinetic differences in the relative fluorescence of chlorophyll *a* observed before and after the inoculation of the *Glycine max* plants with *Sclerotinia sclerotiorum*. Prior to inoculation, the *G. max* plants were bioprimed with rhizopheric bacterial isolates BA15R, BA25R, BA45R, and BA48R, obtained from *Butia acheri*. The data were collected during steps 0 to K = ΔV_OK_ = V_OK_ (pre-pathogen) − V_OK_ (post-pathogen); 0 to J = ΔV_OJ_ = V_OJ_ (pre-pathogen) − V_OJ_ (post-pathogen), and 0 to I = ΔV_OI_ = V_OI_ (pre-pathogen) − V_OI_ (post-pathogen). The red line represents the pre-pathogen kinetic band, and the green line, the post-pathogen band.

**Figure 2:**
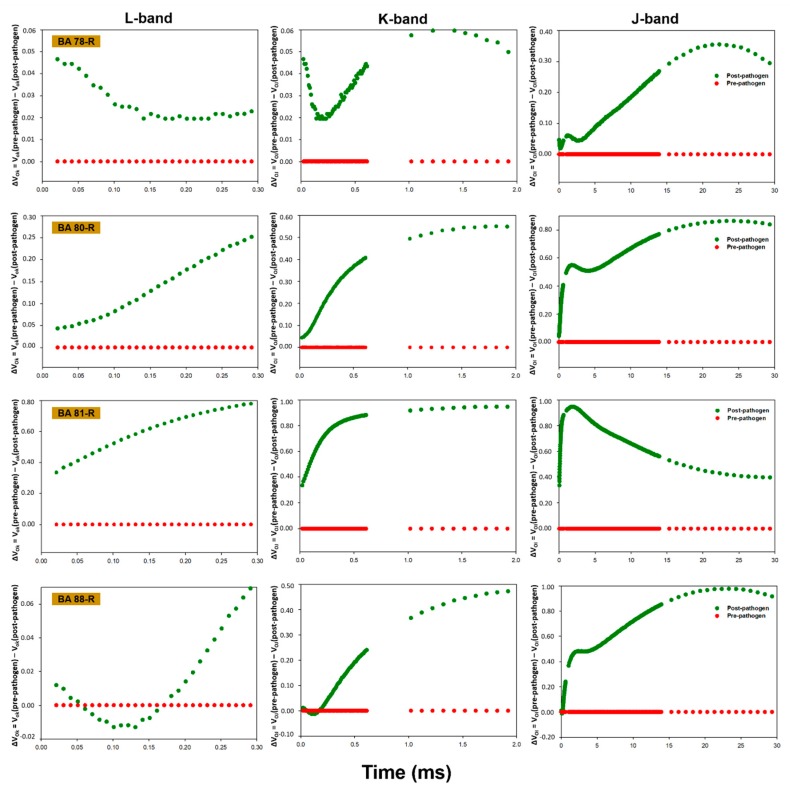
Kinetic differences in the relative fluorescence of chlorophyll *a* observed before and after the inoculation of the *Glycine max* plants with *Sclerotinia sclerotiorum*. Prior to inoculation, the *G. max* plants were bioprimed with rhizopheric bacterial isolates BA78R, BA80R, BA81R, and BA88R, obtained from *Butia acheri*. The data were collected during steps 0 to K = ΔV_OK_ = V_OK_ (pre-pathogen) − V_OK_ (post-pathogen); 0 to J = ΔV_OJ_ = V_OJ_ (pre-pathogen) − V_OJ_ (post-pathogen), and 0 to I = ΔV_OI_ = V_OI_ (pre-pathogen) − V_OI_ (post-pathogen). The red line represents the pre-pathogen kinetic band, and the green line, the post-pathogen band.

**Figure 3:**
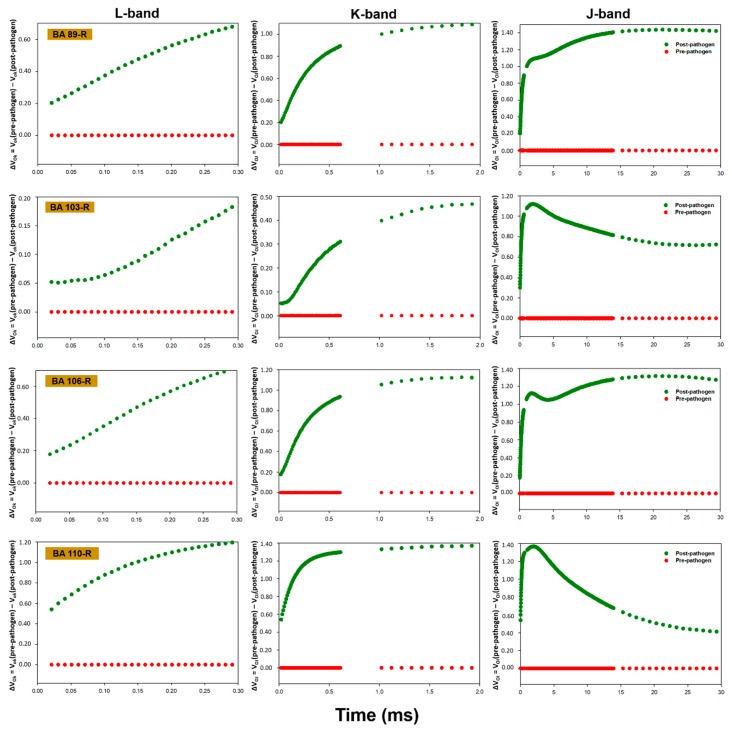
Kinetic differences in the relative fluorescence of chlorophyll *a* observed before and after the inoculation of the *Glycine max* plants with *Sclerotinia sclerotiorum*. Prior to inoculation, the *G. max* plants were bioprimed with rhizopheric bacterial isolates BA89R, BA103R, BA106R, and BA110R, obtained from *Butia acheri*. The data were collected during steps 0 to K = ΔV_OK_ = V_OK_ (pre-pathogen) − V_OK_ (post-pathogen); 0 to J = ΔV_OJ_ = V_OJ_ (pre-pathogen) − V_OJ_ (post-pathogen), and 0 to I = ΔV_OI_ = V_OI_ (pre-pathogen) − V_OI_ (post-pathogen). The red line represents the pre-pathogen kinetic band, and the green line, the post-pathogen band.

**Figure 4:**
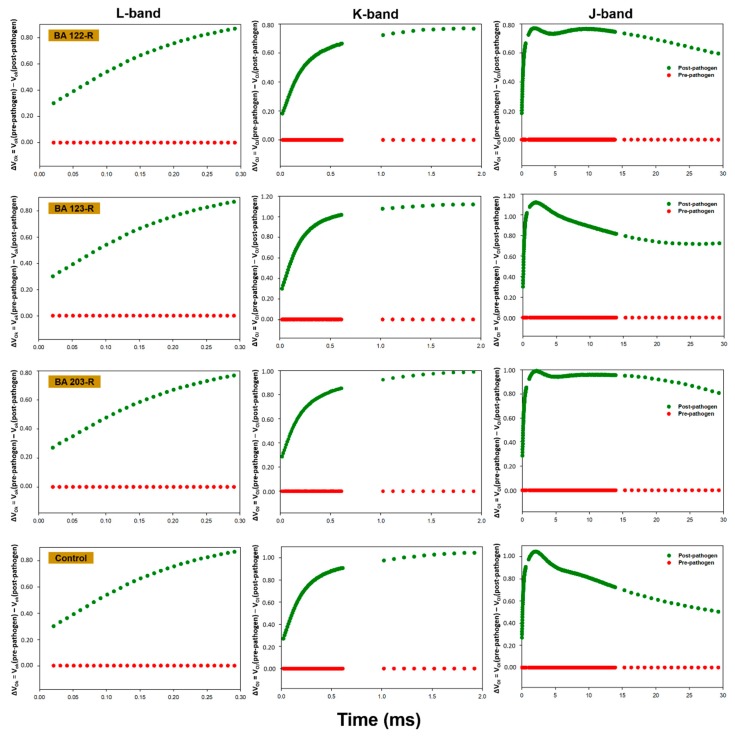
Kinetic differences in the relative fluorescence of chlorophyll *a* observed before and after the inoculation of the *Glycine max* plants with *Sclerotinia sclerotiorum*. Prior to inoculation, the *G. max* plants were bioprimed with rhizopheric bacterial isolates BA122R, BA123R, and BA203R, obtained from *Butia acheri*. The results for the control (not bioprimed) treatment are also shown here. The data were collected during steps 0 to K = ΔV_OK_ = V_OK_ (pre-pathogen) − V_OK_ (post-pathogen); 0 to J = ΔV_OJ_ = V_OJ_ (pre-pathogen) − V_OJ_ (post-pathogen), and 0 to I = ΔV_OI_ = V_OI_ (pre-pathogen) − V_OI_ (post-pathogen). The red line represents the pre-pathogen kinetic band, and the green line, the post-pathogen band.

In general, the plants presented alterations in the pattern of fluorescence transients following inoculation with the phytopathogen, with a reduction in the performance of the photosynthetic apparatus being recorded post-infection in all cases. While the polyphasic OJIP pattern indicated that the plants were photosynthetically active, inoculation with the phytopathogen induced a reduction in the maximum fluorescence (F_m_). The chlorophyll *a* fluorescence data between points O (20 µs) and K (300 µs), between points O (20 µs) and J (2 ms), and between points O (20 µs) and I (30 ms) were normalized and presented as kinetic differences, which permitted the visualization of the L, K and J bands, respectively (Figure 1, Figure 2, Figure 3 and Figure 4). Positive amplitudes were observed in all the treatments in the different bands evaluated kinetically. In the specific case of the plants treated with *Enterobacter* sp. strain BA48R, the kinetics of the initial stage of fluorescence (band L) were similar to that of the plants not inoculated with the pathogen (Figure 1). The only plants in which a negative amplitude was observed during the initial and middle stages of band L were those treated with *B. cereus* strain BA88R (Figure 2).

Low F_v_/F_m_ ratios were recorded (Table 3) prior to inoculation with the phytopathogen in the plants treated with *Enterobacter* sp. strain BA48R (0.767) and *B. cereus* strain BA78R (0.755). As observed in the transient fluorescence curves, the values of this ratio decreased following inoculation with the pathogen in most treatments, indicating a photo-inibitory effect. The values decreased 13.60% in the plants treated with *Enterobacter* sp. strain BA110R, 8.18% in those treated with *B. cereus* strain BA81R, 6.83% in those treated with *B. pumilus* strain BA25R, and 6.49% in the plants treated with *P. putida* strain BA15R.

Following the application of the pathogen, the PI_ABS_ was also reduced in the plants of the majority of the treatments, indicating alterations of the initial stages of the photo-absorption process.

The PI_ABS_ was reduced by 88.75% in the control plants (Table 3), whereas in the plants treated with *Enterobacter* sp. strains BA80R and BA103R and *B. cereus* strain BA88R, the PI_ABS_ levels were maintained (1.321, 1.335 and 1.111, respectively) following inoculation with the pathogen, with no significant differences in comparison with the pre-pathogen levels (1.621, 1.467, and 1.168). The Di_0_/RC ratio followed the opposite pattern in these plants, however, with post-pathogen values of 0.500, 0.500 and 0.602, respectively (Table 3), in comparison with pre-pathogen values of 0.549, 0.553, and 0.623. The plants treated with *Enterobacter* sp. strain BA48R also presented lower post-pathogen Di_0_/RC values in comparison with the pre-pathogen levels, indicating that, despite the stress of the pathogenesis, the energy flow was harnessed more efficiently by the PSII of these plants.

No variation was found among treatments in the general performance of the photosystem prior to the application of the phytopathogen, although the PI_TOTAL_ levels were reduced in the post-pathogen plants. The worst performance (PI_TOTAL_ = 0.040) was recorded in the plants of the control group, reflecting the positive effects of the biopriming with the bacteria on the photosynthetic activity of the plants infected with *S. sclerotiorum*. Curiously, the plants treated with bacterial strains BA49R and BA88R maintained the highest PI_TOTAL_ values following inoculation with the pathogen.

### 3.4. Incidence of Disease

**Figure 5:**
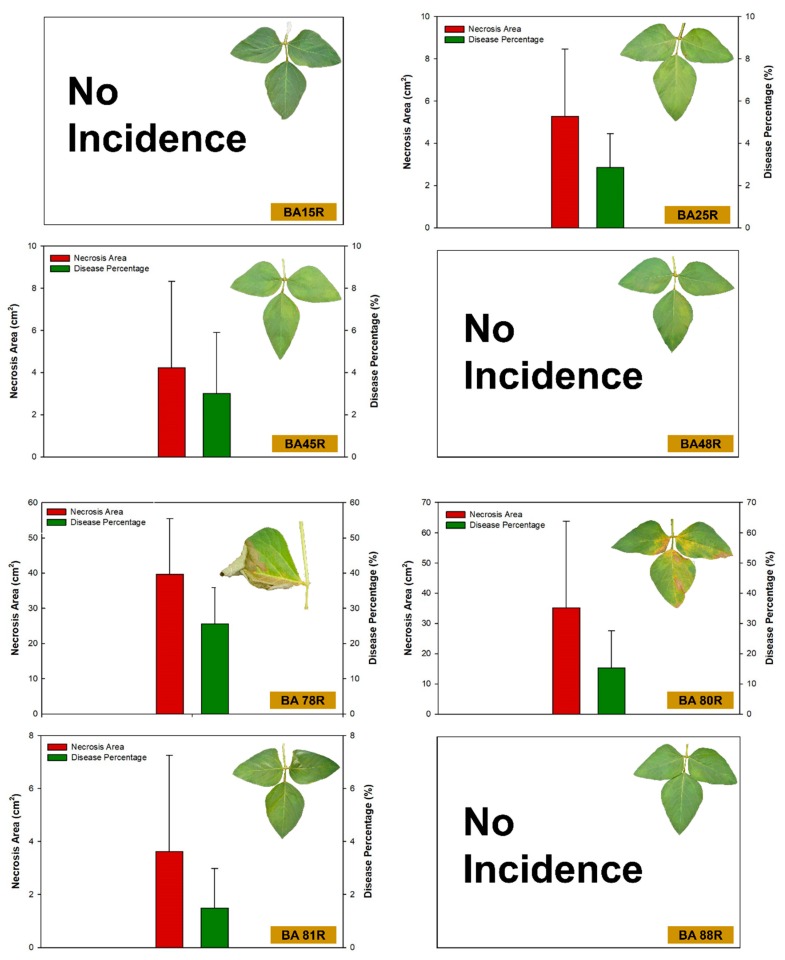
Area of necrosis and percentage diseased leaf cover observed before and after inoculation with *Sclerotinia sclerotiorum* in *Glycine max* plants bioprimed with rhizopheric bacterial isolates of *Butia archeri*.

**Figure 6:**
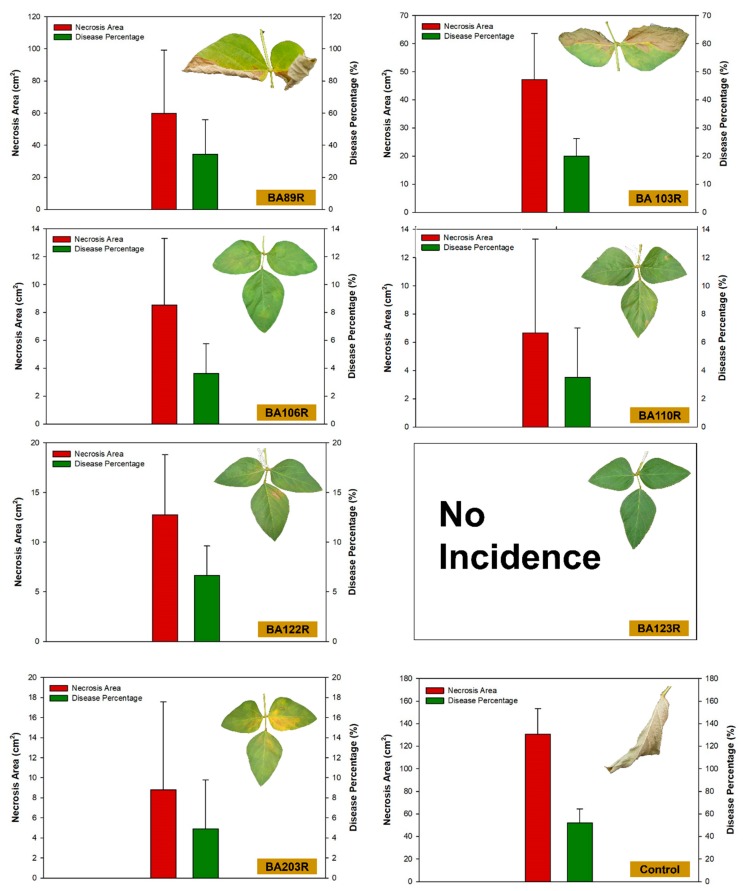
Area of necrosis and percentage diseased leaf cover observed before and after inoculation with *Sclerotinia sclerotiorum* in *Glycine max* plants bioprimed with rhizopheric bacterial isolates of *Butia archeri*.

The soybean plants bioprimed with the bacterial strains BA15R (*P. putida*), BA48R (*Enterobacter* sp.), BA88R (*B. cereus*), and BA123R (*E. asburiae*) did not develop any apparent signs of disease, that is, no tissue necrosis was observed in the leaves inoculated with the pathogen. Small areas of necrosis and low percentages of leaf area with necrosis were found in the plants treated with *B. cereus* strain BA81R (3.52 cm^2^) and *P. cypripedii* strain BA45R (4.22 cm^2^), which represent 1.33% and 3.00% of the leaf area, respectively (Figure 5 and Figure 6). By contrast, the highest levels of tissue damage were observed in the plants that were not bioprimed with bacteria (control treatment), in which the area of diseased tissue was 130.56 cm^2^, corresponding to 51.67% of necrosis.

## 4. Discussion

The BA81R strain of *Bacillus cereus* stimulated the synthesis of total chlorophyll and chlorophyll *a* following inoculation with *S. sclerotiorum*, and the plants treated with the BA81R and BA88R strains of *B. cereus* presented the highest levels of both types of chlorophyll. In the plants treated with the BA88R strain, in fact, the levels of total chlorophyll and chlorophyll *a* recorded following inoculation with the phytopathogen were actually higher than those recorded prior to inoculation. Growth-promoting rhizobacteria may stimulate an increase in the chlorophyll of the plant in the presence of phytopathogens, as observed in the case of the strains of *Pseudomonas*, which increased by 116.57% the chlorophyll content of the leaves of *Cicer arietinum* plants growing on soil contaminated with *Aspergillus* and *Phytophthora* [39]. This indicates that the presence of the bacterial strain BA88R had a positive effect on the metabolism of the plant, reducing the effects of pathogenesis.

A number of studies have described bacteria of the genus *Bacillus* as plant growth promotors due to their production of phytohormones, the solubilization of phosphates or the in vitro antibiosis of phytopathogens, in paired culture systems e.g., [40,41,42,43,44]. In the study of Han et al. [45], however, a strain of *Bacillus subtilis* promoted the growth of *Trifolium repens* L. (Huia cultivar) by regulating, directly or indirectly, the chlorophyll levels of the plant, which is consistent with the pattern observed in some treatments in the present study.

Alternatively, following inoculation with *S. sclerotiorum* the plants bioprimed with *B. cereus* strain BA88R presented the highest NBI value and the lowest flavonoid index, which indicates that these plants did not experience a clear scenario of stress, given that they continued favoring the primary metabolism over the secondary one. The biosynthesis of flavonoids in plants is known to be directly associated with resistance to disease [46] and the anti-fungal action of some flavonoids may be essential to the plant’s capacity to defend itself from the attack of phytopathogens [47]. In this context, a range of flavonoids have been obtained from plant extracts and applied to the control of phytopathogenic fungi e.g., [48,49,50,51], although in the specific case of *S. sclerotiorum*, the bacterial strain BA88R may have been intrinsically antagonistic to the phytopathogen, avoiding the need for the production of large quantities of flavonoids by the soybean plants.

The fluorescence transients were modified in the plants infected with *S. sclerotiorum*, which indicates that this pathogen affects the photosynthetic capacity of the soybean plant. Baghbani et al. [52] demonstrated a similar outcome from the presence of the phytopathogen *Fusarium* sp., which affects the OJIP transients of maize plants. Photosynthesis declines naturally following infection by fungi, generally as a result of the reduction in active leaf area and the loss of chlorophyll [53,54]. This reduction in photosynthetic capacity was demonstrated by the kinetic fluorescence bands, which acquired a positive amplitude following the infection of the plants with *S. sclerotiorum*. In the plants treated with *Enterobacter* sp. strain BA48R, the characteristics of the initial transient stages were similar to those observed in the pre-pathogen plants, although only the plants treated with *B. cereus* strain BA88R presented negative post-pathogen amplitude in the O–K stage, which indicates that this strain may have cushioned the damage caused by the pathogen on the photochemical reduction of quinone A (QA). Even so, the negative amplitudes recorded in the O–J and O–I stages indicate that these plants were unable to maintain their capacity to reduce QA photochemically, and thus did not have the kinetic properties necessary for the reduction/oxidation of the pool of plastoquinones [55]. The negative L band recorded in the plants treated with BA88R indicates that the thylakoid membranes were not affected, and were able to maintain the connectivity between the reaction centers of the PSII. When the L band values are negative, the excitation energy is used more efficiency and the system is more stable [55,56,57].

Following inoculation with the phytopathogen, in fact, photo-inhibitory damage was observed in the plants of most of the study treatments, as indicated by the reduction in the F_V_/F_M_ values. This damage was most accentuated in the plants of the control treatment, in which there was a drastic reduction in both the PI_ABS_ and PI_TOTAL_. In general, previous studies have shown that the presence of pathogens reduces the efficiency of the photosynthesis in the reaction centers. *Euphorbia* plants attacked by the fungus *Uromyces pisi* presented a decrease not only in the F_V_/F_M_ ratio, but also in the PI parameter [53] and Moradi et al. [58] demonstrated the the parameters of chlorophyll fluorescence in cucumber plants sensitive to mildew are affected profoundly, whereas these parameters remain more stable in plants resistant to mildew. In the present study, greater stability was observed in the PI_ABS_ and Di_0_/RC parameters in the plants treated with *Enterobacter* sp. strains BA80R and BA103R and *B. cereus* strain BA88R, with the PI_ABS_ values remaining high following inoculation with the pathogen, and the Di_0_/RC values remaining low. The Di_0_/RC values were also maintained at low levels in the plants inoculated with BA48R. These patterns, which are typical of healthy plants, indicate a high initial absorption of energy and the reduced dissipation of energy by the chlorophylls of the antenna complex.

The PI_TOTAL_ values observed, potential of the plants for the conservation of energy for growth and survival under conditions of stress [56], were higher in biopriming plants than in control, being the best performance verified in plants treated with strains BA48R and BA88R, indicating that these bacteria induced systemic resistance in soybean plants, protecting the performance of the photosynthetic apparatus as a whole. *Bacillus* sp. are known for their relationship to ISR by a jasmonic acid-dependent signaling pathway and miRNAs [59,60].

The leaf area of the control plants was more affected than that of the bacterial treatments, with much larger areas of necrosis. This indicates that the biopriming of the soybean seeds with rhizobacteria was effective for the elimination of the apparent symptoms of the disease. Bacteria associated with plant roots have been shown to have potential for the control of a number of diseases the affect agricultural crops e.g., [61,62,63,64,65]. The bacteria of the genera *Enterobacter* and *Bacillus*, in particular, have attracted considerable attention as the most promising candidates for biocontrol agents of the rhizophere and endorhiza of crop plants [66,67]. Strains of *Enterobacter* and *Bacillus* have already been shown to have antagonistic effects on *S. sclerotiorum* in in vitro assays [68]. Mahartha and Suprapta [69] have reported on the effectiveness of a strain of *Enterobacter* as a suppressant of damping-off in the soybean by *Sclerotium*, while Krishnamoorthy et al. [70] isolated a strain of *B. cereus* capable of reducing the in vitro mycelial growth of *S. sclerotiorum* by 39% in comparison with the control. Rahman et al. [71] demonstrated that isolates of *Bacillus* inhibited the mycelial growth and suppressed the formation of sclerotia in *S. sclerotiorum* during in vitro assays. Deformities and lysis were observed in the cell walls of the mycelia, as well as abnormalities in the apothecium and the failure of the ascospores to germinate.

In the present study, plants protected by *Enterobacter* sp. strain BA48R and *B. cereus* strain BA88R presented no symptoms of infection by *S. sclerotiorum* whatsoever, while those inoculated with *B. cereus* strain BA81R had only weak symptoms, which emphasizes the potential of these bacteria for the biocontrol of this phytopathogen or in the induction of systemic resistance. Similarly, Lozano et al. [72] isolated a strain of *Bacillus* capable of suppressing damping-off, a disease caused by *Phytophthora megasperma* f. sp. *medicaginis*, in alfalfa. The exact biocontrol mechanisms of *Enterobacter* sp. and *B. cereus* remain unclear, however. In an endophytic strain of *Enterobacter*, Taghavi et al. [73] observed the expression of genes related to the synthesis of antimicrobial compounds, such as 4-hydroxybenzoate and 2-phenylethanol, which may protect the plant from attack by both fungi and bacteria. In the specific case of *Bacillus*, a number of studies have indicated that this bacterium may facilitate alterations in the composition of the exudates of the roots, which may have a direct effect on the growth of pathogens [74]. However, extracellular polysaccharides, known as Microbe-Associated Molecular Patterns (MAMPs) [75], may be involved in the biocontrol processes of *B. cereus*.

The results of the present study re-emphasize the importance of the evaluation of fluorescence for the detection of disease-related factors and the stress patterns in crop plants, offering a new approach for the evaluation of the complex interactions of plant-pathogen systems. This approach permitted the identification of the response of the photosynthetic metabolism to the propagation of the pathogens in the plant tissue. The study also proved the effectiveness of the *Enterobacter* sp. BA48R strain and the *B. cereus* BA88R strain for the control of the symptoms provoked by *S. sclerotiorum* in *G. max* plants, reinforcing the potential of these strains for the development of more sustainable agricultural processes.

## 5. Conclusions

When used to bioprime the seeds of the soybean, *Glycine max*, the BA48R and BA88R strains of *B. cereus*, isolated from the dwarf jelly palm, *Butia archeri*, acted as growth-promoting rhizobacteria and provided the best results in terms of the physiological growth of the soybean plants and their resistance to the symptoms of infection by *Sclerotinia sclerotiorum*. High PI_TOTAL_ values and low Di_0_/RC values, as well as an absence of leaf necrosis, were observed in the plants bioprimed with BA48R, although, in general, the bacterium BA88R was the strain that most attenuated the effects of pathogenesis provoked by *S. sclerotiorum*, in particular the loss of chlorophylls and the NBI, which tends to occur as the synthesis of flavonoids increases. Following inoculation with the phytopathogen, the plants treated with this bacterium maintained high values for the parameters PI_TOTAL_ and PI_ABS_, and low values of Di_0_/RC, which are consistent with the conditions found in a healthy plant. This diagnosis was further reinforced by the negative amplitude recorded in the O-K stage of the OJIP transients in the post-inoculation plants, and by the absence of necrosis in their leaves. Overall, then, these bacterial isolates appear to have the most potential for the control of *S. sclerotiorum* in *G. max* plants.

## Figures and Tables

**Table 1:** Chlorophyll (*a*, *b* and total) indices recorded in *Glycine max* plants (Syn 13610^®^ cultivar) bioprimed with rhizospheric bacteria from *Butia archeri* before and after inoculation with the phytopathogen *Scleriotinia sclerotiorum*.

Treatment	Pre-Pathogen			Post-Pathogen		
	Chlorophyll *a*	Chlorophyll *b*	Chlorophyll Total	Chlorophyll *a*	Chlorophyll *b*	Chlorophyll Total
*Pseudomonas putida* (BA15R)	25.73 ± 0.74 * Ca	8.40 ± 0.48 Aa	34.13 ± 1.19 Ba	24.30 ± 1.28 Ba	6.22 ± 0.60 Bb	30.52 ± 1.88 Ba
*Bacillus pumilus* (BA25R)	26.10 ± 0.99 Ca	8.58 ± 0.58 Aa	34.68 ± 1.47 Ba	23.95 ± 0.64 Bb	5.82 ± 0.22 Cb	29.88 ± 0.76 Bb
*Pantoea cypripedii* (BA45R)	22.05 ± 1.84 Ca	6.15 ± 0.87 Aa	28.20 ± 0.67 Ba	22.57 ± 0.45 Ba	6.67 ± 0.24 Ba	29.24 ± 0.67 Ba
*Enterobacter* sp. (BA48R)	25.40 ± 1.08 Ca	8.08 ± 0.36 Aa	33.78 ± 1.36 Ba	21.88 ± 1.60 Cb	5.05 ± 0.60 Cb	26.93 ± 2.19 Cb
*Bacillus cereus* (BA78R)	24.67 ± 1.19 Ca	7.37 ± 0.27 Aa	32.03 ± 1.42 Ba	21.07 ± 0.79 Cb	5.02 ± 0.39 Cb	26.08 ± 1.15 Cb
*Enterobacter* sp. (BA80R)	29.30 ± 0.86 Ba	9.07 ± 0.73 Aa	38.37 ± 1.54 Aa	26.21 0.64 Ab	6.42 ± 0.31 Bb	32.63 ± 0.95 Ab
*Bacillus cereus* (BA81R)	30.63 ± 0.95 Aa	9.85 ± 0.55 Aa	40.48 ± 1.47 Aa	26.93 ± 1.14 Ab	7.42 ± 0.55 Ab	34.02 ± 1.84 Ab
*Bacillus cereus* (BA88R)	24.80 ± 1.18 Ca	7.62 ± 0.37 Aa	32.42 ± 1.60 Ba	26.39 ± 0.66 Aa	6.92 ± 0.13 Ba	33.35 ± 0.76 Aa
*Brevibacillus brevis* (BA89R)	24.42 ± 0.62 Ca	8.00 ± 0.26 Aa	32.42 ± 0.83 Ba	25.77 ± 2.59 Ba	5.61 ± 1.49 Cb	31.38 ± 4.07 Ba
*Enterobacter* sp. (BA103R)	28.35 ± 0.80 Ba	9.07 ± 0.35 Aa	37.42 ± 0.96 Aa	24.53 ± 1.87 Bb	6.22 ± 0.66 Bb	30.75 ± 2.51 Bb
*Enterobacter oryzae* (BA106R)	24.97 ± 0.80 Ca	7.38 ± 0.27 Aa	32.35 ± 0.84 Ba	24.18 ± 1.79 Ba	6.70 ± 0.66 Ba	30.50 ± 2.56 Bb
*Enterobacter* sp. (BA110R)	27.37 ± 1.17 Ba	8.15 ± 0.75 Aa	35.52 ± 1.81 Ba	26.72 ± 1.20 Aa	7.35 ± 0.57 Aa	34.38 ± 1.71 Ab
*Bacillus* sp. (BA122R)	25.68 ± 0.78 Ca	8.15 ± 0.60 Aa	33.83 ± 1.32 Ba	27.03 ± 0.73 Aa	7.68 ± 0.28 Aa	34.72 ± 0.97 Aa
*Enterobacter asburiae* (BA123R)	25.12 ± 1.16 Ca	7.68 ± 0.59 Aa	32.13 ± 2.09 Ba	26.55 ± 0.58 Aa	6.73 ± 0.36 Ba	33.25 ± 0.90 Aa
*Enterobacter asburiae* (BA203R)	25.52 ± 1.21 Ca	8.18 ± 0.61 Aa	33.70 ± 1.75 Ba	23.77 ± 0.82 Ba	6.33 ± 0.56 Bb	30.60 ± 1.68 Bb
Control	29.08 ± 0.92 Ba	9.92 ± 0.31 Aa	39.02 ± 1.15 Aa	21.67 ± 1.23 Bb	5.27 ± 0.44 Cb	26.93 ± 1.67 Bb

The uppercase letters in the columns compare the bacterial treatments, while the lowercase letters in the lines compare the pre- and post-pathogen groups. * = standard error of the mean. The means followed by different letters in either the lines or columns are significantly different from each other, based on the Scott Knott test (5% probability).

**Table 2:** Nitrogen balance (NBI), flavonoid (FLAV) and anthocyanin indices (ANTH) recorded in *Glycine max* plants (Syn 13610^®^ cultivar) bioprimed with rhizospheric bacteria from *Butia archeri* before and after inoculation with the phytopathogen *Scleriotinia sclerotiorum*.

Treatment	Pre-Pathogen			Post-Pathogen		
	NBI	FLAV	ANTH	NBI	FLAV	ANTH
*Pseudomonas putida* (BA15R)	21.83 ± 0.14 * Ba	1.23 ± 0.03 Ab	0.24 ± 0.01 Ab	13.50 ± 3.26 Bb	1.56 ± 0.06 Aa	0.32 ± 0.04 Aa
*Bacillus pumilus* (BA25R)	21.90 ± 1.74 Ba	1.21 ± 0.08 Ab	0.28 ± 0.03 Aa	13.13 ± 1.98 Bb	1.56 ± 0.08 Aa	0.30 ± 0.01 Aa
*Pantoea cypripedii* (BA45R)	19.53 ± 1.48 Ba	1.30 ± 0.07 Ab	0.27 ± 0.03 Ab	14.13 ± 0.30 Bb	1.49 ± 0.05 Ba	0.31 ± 0.01 Aa
*Enterobacter* sp. (BA48R)	18.83 ± 1.00 Ba	1.30 ± 0.06 Ab	0.25 ± 0.01 Ab	13.03 ± 2.98 Bb	1.52 ± 0.02 Ba	0.32 ± 0.03 Aa
*Bacillus cereus* (BA78R)	21.17 ± 1.90 Ba	1.23 ± 0.09 Ab	0.23 ± 0.01 Ab	13.30 ± 0.25 Bb	1.40 ± 0.10 Ba	0.33 ± 0.01 Aa
*Enterobacter* sp. (BA80R)	23.87 ± 0.81 Ba	1.16 ± 0.04 Bb	0.26 ± 0.01 Ab	14.43 ± 2.82 Bb	1.53 ± 0.13 Ba	0.32 ± 0.03 Aa
*Bacillus cereus* (BA81R)	27.83 ± 2.94 Aa	1.13 ± 0.10 Bb	0.22 ± 0.00 Ab	14.63 ± 0.92 Bb	1.43 ± 0.06 Ba	0.31 ± 0.01 Aa
*Bacillus cereus* (BA88R)	23.30 ± 1.72 Ba	1.20 ± 0.05 Ab	0.27 ± 0.01 Ab	18.87 ± 2.87 Ab	1.39 ± 0.28 Ba	0.29 ± 0.02 Ba
*Brevibacillus brevis* (BA89R)	19.87 ± 1.22 Ba	1.23 ± 0.03 Ab	0.23 ± 0.01 Ab	15.20 ± 3.80 Bb	1.44 ± 0.11 Ba	0.31 ± 0.04 Aa
*Enterobacter* sp. (BA103R)	29.10 ± 0.81 Aa	1.03 ±0.03 Bb	0.24 ± 0.01 Ab	15.83 ± 2.80 Bb	1.39 ± 0.07 Ba	0.31 ± 0.03 Aa
*Enterobacter oryzae* (BA106R)	28.90 ± 1.71 Aa	1.03 ±0.06 Ba	0.26 ± 0.01 Ab	13.27 ± 1.51 Bb	1.08 ± 0.06 Ba	0.31 ± 0.02 Aa
*Enterobacter* sp. (BA110R)	23.73 ± 1.50 Ba	1.23 ± 0.07 Ab	0.25 ± 0.03 Ab	15.60 ± 1.11 Bb	1.39 ± 0.08 Ba	0.30 ± 0.00 Aa
*Bacillus* sp. (BA122R)	24.13 ± 1.49 Ba	1.18 ± 0.03 Ab	0.24 ± 0.01 Ab	14.73 ± 2.29 Bb	1.45 ± 0.10 Ba	0.30 ± 0.02 Aa
*Enterobacter asburiae* (BA123R)	24.40 ±1.32 Ba	1.21 ± 0.06 Ab	0.23 ± 0.00 Ab	15.87 ± 2.01 Bb	1.57 ± 0.06 Aa	0.28 ± 0.02 Ba
*Enterobacter asburiae* (BA203R)	24.00 ± 0.17 Ba	1.21 ± 0.04 Ab	0.24 ± 0.02 Ab	14.60 ± 1.81 Bb	1.46 ± 0.08 Ba	0.31 ± 0.02 Aa
Control	23.03 ± 1.77 Ba	1.25 ± 0.06 Ab	0.22 ± 0.01 Ab	14.00 ± 0.85 Bb	1.48 ± 0.05 Ba	0.29 ± 0.02 Ba

The uppercase letters in the columns compare the bacterial treatments, while the lowercase letters in the lines compare the pre- and post-pathogen groups. * = standard error of the mean. The means followed by different letters in either the lines or columns are significantly different from each other, based on the Scott Knott test (5% probability).

**Table 3:** Mean F_v_/F_m_, PI_ABS_, and Di_0_/RC values recorded in *Glycine max* plants (Syn 13610^®^ cultivar) bioprimed with rhizospheric bacteria from *Butia archeri* before and after inoculation with the phytopathogen *Scleriotinia sclerotiorum*.

Treatment	Pre-Pathogen				Post-Pathogen			
	F_V_/F_M_	PI_ABS_	Di_0_/RC	PI_TOTAL_	F_V_/F_M_	PI_ABS_	Di_0_/RC	PI_TOTAL_
*Pseudomonas putida* (BA15R)	0.801 ± 0.000 * Aa	1.558 ± 0.085 Ba	0.549 ± 0.016 Cb	1.810 ± 0.053 Aa	0.749 ± 0.015 Bb	0.540 ± 0.078 Cb	0.759 ± 0.059 Ba	0.770 ± 0.005 Bb
*Bacillus pumilus* (BA25R)	0.805 ± 0.008 Aa	1.635 ± 0.200 Ba	0.542 ± 0.038 Cb	1.720 ± 0.138 Aa	0.750 ± 0.009 Bb	0.540 ± 0.034 Cb	0.762 ± 0.033 Ba	0.880 ± 0.043 Bb
*Pantoea cypripedii* (BA45R)	0.794 ± 0.001 Aa	1.229 ± 0.106 Ca	0.579 ± 0.012 Ca	1.540 ± 0.155 Aa	0.762 ± 0.033 Ba	0.764 ± 0.327 Bb	0.701 ± 0.153 Ba	0.100 ± 0.413 Cb
*Enterobacter* sp. (BA48R)	0.767 ± 0.004 Ca	0.889 ± 0.097 Da	0.720 ± 0.020 Aa	1.660 ± 0.167 Aa	0.768 ± 0.014 Ba	0.793 ± 0.221 Ba	0.600 ± 0.052 Cb	1.280 ± 0.107 Ab
*Bacillus cereus* (BA78R)	0.755 ± 0.003 Da	0.694 ± 0.093 Da	0.764 ± 0.033 Aa	2.040 ± 0.408 Aa	0.758 ± 0.026 Ba	0.871 ± 0.225 Ba	0.755 ± 0.129 Ba	1.240 ± 0.080 Ab
*Enterobacter* sp. (BA80R)	0.798 ± 0.001 Ab	1.42 ± 0.041 Ba	0.549 ± 0.009 Ca	2.060 ± 0.077 Aa	0.807 ± 0.001 Aa	1.321 ± 0.144 Aa	0.500 ± 0.012 Cb	1.190 ± 0.097 Ab
*Bacillus cereus* (BA81R)	0.807 ± 0.001 Aa	1.621 ± 0.085 Ba	0.501 ± 0.010 Cb	2.240 ± 0.050 Aa	0.741 ± 0.014 Bb	0.478 ± 0.062 Cb	0.787 ± 0.078 Ba	1.100 ± 0.191 Ab
*Bacillus cereus* (BA88R)	0.787 ± 0.001 Ba	1.168 ± 0.023 Ca	0.623 ± 0.011 Ba	2.000 ± 0.157 Aa	0.785 ± 0.006 Aa	1.111 ± 0.028 Aa	0.602 ± 0.012 Cb	1.300 ± 0.028 Ab
*Brevibacillus brevis* (BA89R)	0.786 ± 0.002 Ba	1.231 ± 0.034 Ca	0.596 ± 0.006 Ba	2.750 ± 0.510 Aa	0.784 ± 0.006 Aa	0.916 ± 0.105 Ba	0.594 ± 0.034 Ca	1.200 ± 0.094 Ab
*Enterobacter* sp. (BA103R)	0.801 ± 0.006 Aa	1.467 ± 0.061 Ba	0.553 ± 0.021 Ca	1.750 ± 0.069 Aa	0.804 ± 0.004 Aa	1.355 ± 0.067 Aa	0.500 ± 0.017 Cb	1.020 ± 0.017 Ab
*Enterobacter oryzae* (BA106R)	0.816 ± 0.001 Aa	2.149 ± 0.057 Aa	0.472 ± 0.008 Ca	2.620 ± 0.081 Aa	0.809 ± 0.008 Aa	1.335 ± 0.232 Ab	0.504 ± 0.024 Ca	1.170 ± 0.071 Ab
*Enterobacter* sp. (BA110R)	0.801 ± 0.001 Aa	1.500 ± 0.080 Ba	0.536 ± 0.011 Cb	2.370 ± 0.152 Aa	0.692 ± 0.029 Cb	0.272 ± 0.158 Cb	1.069 ± 0.162 Aa	0.280 ± 0.264 Cb
*Bacillus* sp. (BA122R)	0.778 ± 0.012 Ba	0.962 ± 0.245 Da	0.637 ± 0.048 Bb	1.900 ± 0.073 Aa	0.738 ± 0.007 Ba	0.354 ± 0.074 Cb	0.829 ± 0.035 Ba	0.790 ± 0.024 Bb
*Enterobacter asburiae* (BA123R)	0.802 ± 0.001 Aa	1.579 ± 0.142 Ba	0.536 ± 0.022 Ca	1.830 ± 0.026 Aa	0.782 ± 0.007 Aa	0.993 ± 0.114 Bb	0.618 ± 0.047 Ba	1.190 ± 0.012 Ab
*Enterobacter asburiae* (BA203R)	0.800 ± 0.004 Aa	1.645 ± 0.049 Ba	0.559 ± 0.016 Ca	1.700 ± 0.027 Aa	0.764 ± 0.011 Ba	0.807 ± 0.124 Bb	0.706 ± 0.063 Ba	0.820 ± 0.019 Bb
Control	0.781 ± 0.009 Ba	1.156 ± 0.052 Ca	0.630 ± 0.052 Bb	1.430 ± 0.174 Aa	0.802 ± 0.005 Aa	0.130 ± 0.013 Cb	0.689 ± 0.079 Ba	0.040 ± 0.172 Db

The uppercase letters in the columns compare the bacterial treatments, while the lowercase letters in the lines compare the pre- and post-pathogen groups. * = standard error of the mean. The means followed by different letters in either the lines or columns are significantly different from each other, based on the Scott Knott test (5% probability).
